# Using *Clinical Evidence* in a National Continuing Medical Education Program in Italy

**DOI:** 10.1371/journal.pmed.0040113

**Published:** 2007-05-22

**Authors:** Lorenzo Moja, Ivan Moschetti, Alessandro Liberati, Roberto Manfrini, Christian Deligant, Roberto Satolli, Antonio Addis, Nello Martini, Pietro Dri

## Abstract

In an effort to ensure that physicians have access to reliable evidence on drug effectiveness and safety, the Italian Drug Agency launched a program to disseminate independent information.

Interest in evidence-based medicine (EBM) is growing in Italy, although its impact upon health policies and clinical practice is unclear. Rather than getting health information from unbiased evidence-based sources, doctors in Italy still rely heavily upon the pharmaceutical industry for their information needs. For example, a recent survey showed that general practitioners receive 11 visits per week by pharmaceutical sales representatives [1]. The study suggested that this information is considered complete and sufficiently reliable by many doctors.

In an effort to ensure that all physicians have access to valid and reliable evidence on drug effectiveness and safety, the Italian Drug Agency (AIFA) launched a program to disseminate independent and unbiased information. The agency did this by translating *Clinical Evidence*, a compendium of the best available evidence on treating a wide range of common conditions (Box 1), into Italian and distributing it freely. By 2006, the fourth Italian edition (based on *Clinical Evidence* volume 14) had been published. The online Italian version of *Clinical Evidence* is freely available to all 248,000 doctors in practice in Italy.

In 1999, the first free distribution of 50,000 copies of *Clinical Evidence* was assessed through a survey exploring doctors' judgement of its validity, relevance, and usability. Results showed that the compendium had been well received, and confirmed doctors' preference for problem-driven information and the key role of a strong endorsement from health authorities for its implementation [2].

**Box 1. *Clinical Evidence***

*Clinical Evidence* (http://www.clinicalevidence.com) has specific features that make it different from both traditional textbooks and practice guidelines [19]:

- Its contents are driven by practical questions rather than by the availability of evidence.
- It aims not to make recommendations but to inform based on the best available evidence.
- It highlights rather than hides gaps in research evidence.
- It is continuously updated.

According to its Web site, *Clinical Evidence* “describes the best available evidence from systematic reviews, RCTs [randomised controlled trials], and observational studies where appropriate, and if there is no good evidence it says so.”

A compulsory system of continuing medical education (CME) for all health professionals was introduced in Italy in 1998, based on credits awarded for time spent on educational activities. The more traditional form of acquiring CME is to attend lectures and conferences; it is much more rare for doctors to be exposed to small group interactive events. In order to maximise the effectiveness of the financial commitment for disseminating *Clinical Evidence*, and to speed up the diffusion of EBM, AIFA sponsored a free-access e-learning system, based on *Clinical Evidence*, called ECCE (the Italian acronym for Continuing Education *Clinical Evidence*).

## ECCE: An e-Learning CME Program

ECCE is an e-learning CME tool that uses interactive clinical vignettes based on chapters in *Clinical Evidence* and a predefined sequence of questions. The vignettes reflect real-life circumstances as seen by an ordinary general practitioner in everyday practice. Whilst the primary target group of ECCE is general practitioners, many vignettes are also relevant to specialists.

Each vignette has a narrative with events presented in chronological order: the history evolves with new insights from diagnostic tests or additional information reported by the patient. Users progress from step to step by overcoming a question/answer decision system. Box 2 gives an example of a vignette from ECCE.

**Box 2. Example of An ECCE Vignette**

We present the first step of the headache (chronic tension-type) vignette and the related question. The vignette was developed from the chapter Headache (chronic tension-type), in Clinical Evidence [20].

Margaret says to her family doctor: “This time I didn't come for me, but to talk about Rachel, my 25-year-old daughter. As you probably remember, she got married last year; unfortunately, she doesn't seem to get along well with her husband... Anyway, the other day she told me that in the past few months she has often suffered from headache; I'm quite worried about that, you know what I have been through...” The doctor remembers very well that many years before, Margaret, one of his first patients, was always complaining about her headache, a pain that tormented her all the time and was not relieved by analgesics. He tells Margaret to come back with her daughter. After a few days the two women are in the doctor's office. Rachel says: “This headache is killing me. I have it every day now, sometimes with nausea. And I don't want to take analgesics any more, they don't do me any good”. She describes her pain as a bilateral, tight, “band-like” discomfort: “My head feels as if it is in a vice. Following the advice of a friend of mine I have also tried to take some drops of a benzodiazepine; but the headache didn't go away, and I felt drowsy and light headed”. Rachel looks very pale, tired and tense. Examining her, the doctor doesn't find anything abnormal; he suspects a chronic tension-type headache, and prescribes a battery of blood tests.

**Question:** According to the studies identified by *Clinical Evidence*, in Rachel's case benzodiazepines:

- may be very effective, but only in the short term
- may be effective, but only in the long term
- may be useful only in people with very severe headache
- may induce a modest short term improvement, but are often associated with adverse effects
- are contraindicated

**The correct answer is:** In Rachel's case, benzodiazepines may induce a modest short term improvement, but are often associated with adverse effects.

The response option includes one or more correct answers for each question, and other possible responses as distractors. Users gain credits upon completing all steps as long as they reach a score above 80% of the maximum total score. Vignettes provide one or two credits depending on the number of questions in the vignette. During the first year we posted 120 vignettes.

ECCE has all the standard advantages of e-learning. Users select what they wish to learn, when and at what pace. The system is easy to use and works with basic computer requirements (e.g., low speed connections). The contents of *Clinical Evidence* can be read on screen or printed and interactively managed along the steps of each vignette. The system tracks learning content and the learner's progress. Eight vignettes translated in English are available at http://ecce-gb.fad-ecm.it (registration required).

ECCE became accessible to all physicians in March 2005 after a pilot period. Here we present the results of the first year of usage (until February 2006).

## Doctors' Use of ECCE

**Figure 1:**
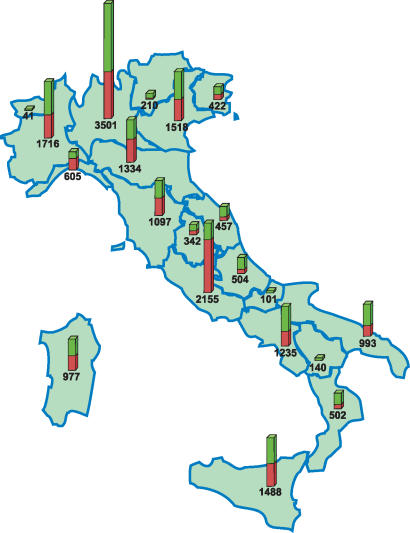
Doctors Using ECCE by Region Red: urban areas; green: rural areas.

In one year, 19,340 doctors voluntarily subscribed to ECCE (7.8% of all Italian practicing doctors) and around 93% logged in completing at least one vignette (Table 1). Almost one quarter (4,429, or 22.9%) were general practitioners (Table 2). The median age of the users was 50 years (interquartile range 40 to 56). Over half (52%) of users were based in rural areas, and there was regional variation in use: Sardinia had the highest rate of use (9.87% of all resident doctors) while Campania had the lowest (3.94%), and there was a modest north–south usage gradient (Figure 1).

**Table 1:**
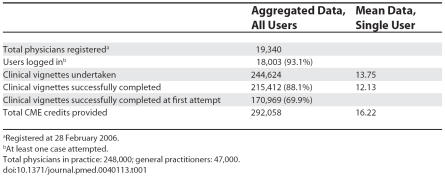
Use of ECCE by Registered Doctors and Average Use by Single User

**Table 2:**
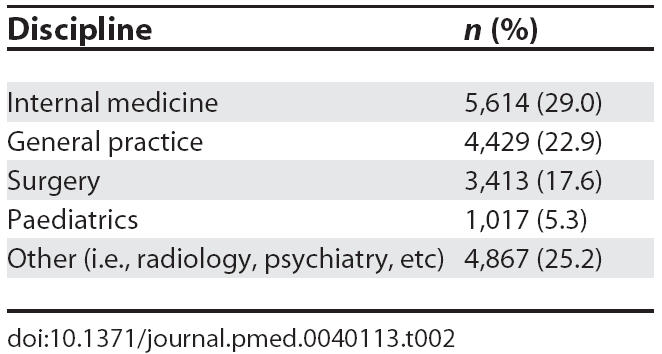
Doctors' Professional Profiles

Doctors used ECCE throughout the entire day, with early morning and late evening being the most popular time slots. Altogether 215,412 vignettes have been completed and 292,058 credits awarded. The average number of completed vignettes for a single user was 13.75 (median 50, interquartile range 28–101), with a corresponding average credit of 16.22. There were 3,468 doctors who obtained 25 or more credits (30.2% of those who acquired at least one credit), exceeding the mandatory ministerial requirement (24 credits in 2005 for distance learning).

The top five accessed vignettes were appendicitis, atrial fibrillation, herpes zoster, paracetamol poisoning, and acute low back pain. The easiest vignette to be solved had a success rate of 93.91% at first attempt, while the average success rate was 79.19% and the hardest (“statistically advanced”) case had a 39.10% success rate. Mean time to interactively complete a vignette was nine minutes for one credit history and 18 minutes for two credit histories. Before completing the vignette, doctors are supposed to peruse the related chapter in *Clinical Evidence* (which is assumed to take five minutes per page).

During the first year the cost per credit supplied through ECCE was 2.4 Euros (including all direct costs: license to BMJ Publishing Group to reproduce *Clinical Evidence*, translation of the fourth edition into Italian, development of the e-learning CME system and its management and technological platform). The 2.4 Euros excludes the costs of printing *Clinical Evidence* as well as indirect costs. All costs are tax free. Discounting the costs related to translation of *Clinical Evidence* and to the start-up of the platform, the cost per credit was 0.7 Euros.

## Users' Survey

At the end of each vignette doctors were asked—using an online questionnaire—to provide comments about their experience solving ECCE's cases (75.1% response rate). ECCE's vignettes were well received (Table 3): more than 90% of users considered them relevant and appropriate for educational purposes, and out of 21,589 free-text feedback messages, 17,902 (82.9%) were positive.

**Table 3:**
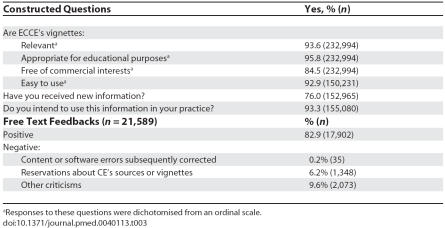
Respondents' Opinions about ECCE

Overall, these opinions were largely consistent with what emerged from three focus groups run in northern, southern, and central Italy. The 26 clinicians who participated felt that ECCE was well representative of their clinical practice and that the information conveyed was reliable and relevant, despite the absence of evidence on how to make diagnoses (*Clinical Evidence* focuses on treatment, not diagnosis). The clinicians complained that reading the full chapter of *Clinical Evidence* was tedious, but appreciated the format of the clinical vignette, which allowed them to switch from passive reading to an active learning exercise.

## Discussion

The large number of subscribers to ECCE suggests that this CME programme is meeting an educational need. However, this number still represents only a small proportion (7.8%) of all practising doctors. But there has been limited promotion of ECCE, involving only a small number of medical journal adverts, and the program is still in its infancy.

### Limitations

This study has several limitations. The first limitation is selection bias: there may have been an over-representation in our sample of doctors with a generally positive attitude toward EBM and with greater computer skills. ECCE has probably filled the gap that still exists between a growing demand for reliable and independent critical appraisal and a limited offering [3]. Thus the doctors in our survey could be classified as early adopters of an evidence-based innovation and may have been different in key ways (i.e., having a positive perception of EBM) from the rest of Italian doctors [4].

A second limitation relates to the CME structural factor (i.e., mandatory requirement): we cannot rule out the possibility that some doctors intentionally used ECCE with the aim of collecting as many credits as possible. This “opportunistic use” was also highlighted during the focus groups. However, at least 30.2% of doctors exceeded the CME mandatory requirement, after which they were presumably using ECCE to challenge their own knowledge and competence.

The last two limitations deal with the innovation itself (i.e., ECCE). Does the high success rate of the vignettes mean that ECCE was unable to discriminate poor performers from better ones? The average success rate was close to 80% (considered a moderately easy test). The test discrimination capability could be biased towards middle achievers, an attribute which could be seen negatively. We think that this is not negative itself: innovations that are not difficult to do (low complexity) and easy to try (high trialability) maximise their perceived attraction and adoption [5].

A fourth limitation is that positive results may reflect physician competence more than appropriate clinical practice. While changing provider behaviour could be seen as the final step of the innovation decision process [6], it should be preceded by change in other dimensions, particularly knowledge [7,8]. If ECCE has any such effect, it influences knowledge and competency in using EBM. While the recognition of the teaching properties of case histories is not new [9–11], in a recent study vignettes' scores appeared to be highly correlated to physician practice in outpatient settings and were a valid overall measure of the process of care provided [12]. Furthermore, one of the greatest barriers to reading *Clinical Evidence* was boredom. The use of written case simulation seemed to transform the passive reading into a more interactive experience in which doctors searched for the right piece of information to be applied in specific situations.

### The evidence-based innovation

AIFA supported the diffusion of *Clinical Evidence*, a user-friendly medical resource free of conflicts of interest. This approach is different from that of other countries, which supported, for example, free access to Cochrane systematic reviews [13]. Although the systematic reviews in *Clinical Evidence* and the Cochrane Library are related [14], *Clinical Evidence* was thought by policy makers to be more useful in daily practice by answering common and important clinical questions using clear summaries. Recognising the value of independent information, AIFA is attempting to balance a context where pharmaceutical companies have been the only information drivers for many years. The adoption of high quality information on EBM enhances doctors' mastery of information, emphasising sources that are high in relevance and validity, and that do not require a huge amount of time to access [15].

In 2005 the cost for each credit provided through ECCE was less then 2.5 Euros. In 2006, maintaining the same patterns of usage, the estimated cost will be 0.7 Euros/credit, a reasonable effort considering that in 2004 the average cost of each CME credit in Emilia-Romagna, a region that monitored this expenditure, was 144.0 Euros [16]. Furthermore, this investment in independent information pales in comparison to the 3 billion Euros spent by pharmaceutical companies on marketing their drugs in Italy in 2004, an investment of about 8,000 Euros for each doctor [17].

## Conclusion

Cultural and policy changes are brought about by a complex interaction of social, economic, and political factors, including the influence of thought leaders. In 2001 Smith and Chalmers wrote: “Universal free access to an integrated information resource built from the Cochrane Library, *Clinical Evidence*, and the metaRegister of Controlled Trials would go some way to reducing the inequities in access to information for improving health care” [18]. Italy is now starting to move in this direction.

## Abbreviations

CME: continuing medical education
EBM: evidence-based medicine
